# Use of Menthol Cigarettes, Smoking Frequency, and Nicotine Dependence Among US Youth

**DOI:** 10.1001/jamanetworkopen.2022.17144

**Published:** 2022-06-06

**Authors:** Eric C. Leas, Tarik Benmarhnia, David R. Strong, John P. Pierce

**Affiliations:** 1 Herbert Wertheim School of Public Health and Human Longevity Science, University of California, San Diego, La Jolla; 2 Scripps Institution of Oceanography, University of California, San Diego, La Jolla

## Abstract

**Importance:**

Menthol may make cigarette smoke less aversive and could lead youth to smoke more frequently and become more dependent on nicotine.

**Objective:**

To assess the association of menthol use with cigarette smoking frequency and nicotine dependence (ND) among youth cigarettes users.

**Design, Setting, and Participants:**

This cohort study used a pooled sample of cohorts of US youth (aged 12-17 years at their baseline and follow-up interviews) in the Population Assessment of Tobacco and Health Study (September 2013 to November 2019). Household interviews were conducted using audio computer-assisted self-interviews. US youth who were past-30-day cigarette smokers at any of wave 2 through wave 5, completed the preceding survey (eg, wave 2 past-30-day cigarette smokers completed wave 1), and indicated whether they used menthol cigarettes were eligible. The association of menthol use with smoking frequency and ND was estimated using inverse probability of treatment weighting adjusted mean differences (aMDs), adjusted risk ratios (aRRs), and corresponding 95% CIs. Data were analyzed from December 2021 to March 2022.

**Exposures:**

Menthol use and transitions in use (switching to or from menthol cigarettes).

**Main Outcomes and Measures:**

Main outcomes were the number of days smoked in the past 30 days, the risk of frequent smoking (smoking ≥20 days in the past 30 days), and symptoms of ND.

**Results:**

There were a total of 1492 observations among 1096 US youth cigarette smokers, of whom 49.4% were female, 67.2% were non-Hispanic White, and 28.7% were aged 12 to 14 years at their baseline survey (all percentages are weighted). Among those who switched from not smoking to smoking (61% of the sample), menthol use was associated with smoking on 3.1 additional days (aMD; 95% CI, 1.9 to 4.2 days), 59% higher risk of being a frequent smoker (aRR, 1.59; 95% CI, 1.23 to 2.06), and 10% higher ND scores (aMD, 1.09; 95% CI, 1.02 to 1.17). Switching from smoking menthol to smoking nonmenthol (vs maintaining menthol use) was associated with smoking on 3.6 fewer days (aMD; 95% CI, −6.3 to −0.9 days) and 47% lower risk of being a frequent smoker (aRR, 0.68; 95% CI, 0.50 to 0.92).

**Conclusions and Relevance:**

These findings suggest that the addition of menthol to cigarettes is associated with increased smoking frequency and ND among US youth.

## Key Points

**Question:**

Do youth menthol cigarette smokers smoke more frequently and display greater symptoms of nicotine dependence than youth nonmenthol cigarette smokers?

**Findings:**

In this population-based pooled cohort study including 1492 observations of 1096 youth cigarette smokers (aged 12-17 years at their baseline and follow-up interviews), menthol use was associated with smoking more frequently and with having higher nicotine dependence scores, but switching from menthol to nonmenthol cigarettes was associated with reduced smoking frequency and dependence.

**Meaning:**

These findings suggest that the addition of menthol to cigarettes could increase smoking frequency and nicotine dependence among youth.

## Teaser

This cohort study assesses the association of menthol cigarette use with smoking frequency and nicotine dependence among youth cigarettes users.

## Introduction

In April 2021, the US Food and Drug Administration (FDA) committed to proposing new product standards that could prohibit the manufacturing and distribution of menthol cigarettes in the US.^1^ Their next step is scheduled for spring 2022,^2^ and former FDA Commissioner Scott Gottlieb recently described the agency’s feedback on the experience as “the most intense political fight that I had while I was at the agency on tobacco.”^3^ Although the FDA’s own advisory committee recommended banning menthol from cigarettes in 2011,^4^ the tobacco industry and other stakeholders have consistently argued that available studies do not justify flavor restrictions.^5^ To guide deliberations, it will be critical to continue to strengthen the evidence base on the public health impacts of menthol cigarettes.

One public health concern prompting recommendations to ban menthol cigarettes is that menthol may alter the biochemical profile of cigarette smoke in a manner that could increase smoking frequency and nicotine dependence (ND), particularly among new smokers.^4^ There are at least 2 biological mechanisms by which menthol could have such an impact.^6^ First, menthol has local analgesic and anesthetic properties that suppress tissue irritation and, when added to cigarettes, can allow for deeper and longer inhalation of smoke, potentially resulting in a greater absorption of nicotine per cigarette puff.^7,8,9^ This process may be most critical during smoking initiation (when smoking is most aversive^10^), which typically occurs before the age of 18 years.^11^ In addition, neuroscientific evidence suggests that exposure to nicotine and menthol in combination, compared with nicotine alone, results in a greater number of nicotinic acetylcholine receptors and more dopaminergic activation in the ventral tegmental area, both potentially leading to greater reinforcement from nicotine and more reward-seeking behaviors.^12,13^

Epidemiological studies^14,15^ also support the biological plausibility that the addition of menthol to cigarettes may increase smoking frequency and dependence. For example, a recent review of observational research on menthol use stated that the evidence was sufficient to conclude that “youth menthol smokers report greater levels of nicotine dependence than youth non-menthol smokers” and that “initiation with menthol cigarettes facilitates progression to established use in young smokers”.^14^ However, each of these conclusions relied primarily on evidence from cross-sectional studies, with the only longitudinal study reported being conducted on a sample that was not nationally representative, potentially reducing the generalizability of the results.^15^

The launch of 2 FDA-funded nationally representative longitudinal cohort studies^16,17^ has provided the opportunity to fill this gap in the literature with robust longitudinal analyses of menthol use. Nonnemaker et al^16^ analyzed the Evaluation of Public Education Campaign on Teen Tobacco Cohort Study and found an association between using menthol cigarettes and progression from experimental to established current smoking among youth. Using the Population Assessment of Tobacco and Health (PATH) Study, Villanti et al^17^ identified a prospective association between the first use of a menthol cigarette at wave 1 of the PATH Study and subsequent cigarette use at wave 2 in all age groups. However, when Villanti et al^18^ extended this analysis to waves 1 through 4, evidence of an association between first use of menthol and subsequent smoking was found only for young adults, not adolescents, and there was no evidence of an association between menthol use and subsequent ND.

The primary objective of the current cohort study was to further strengthen the evidence base for the association between menthol use and youth smoking frequency and ND by exploiting the multiwave design of the PATH Study and by using an advanced analytical technique known as inverse probability of treatment weighting (IPTW). Our design allowed us to assess the associations of changes in smoking status and switching to and from menthol with frequency of use and dependence. We hypothesized that switching from smoking nonmenthol to smoking menthol cigarettes would be associated with increased smoking frequency and dependence, whereas switching from smoking menthol to smoking nonmenthol cigarettes would be associated with decreased smoking frequency and dependence. We also hypothesized that among those who went from not smoking at one wave to smoking at the next, use of menthol at the subsequent wave would be associated with increased smoking frequency and dependence.

## Methods

### Data Source and Analytical Sample

Our data incorporate waves 1 through 5 of the youth surveys available in the public use files of the PATH Study. Wave 1 data collection (September 2013 to December 2014) resulted in a nationally representative sample of 13 651 youths aged 12 to 17 years (74.0% response rate) and 7207 youths aged 9 to 11 years who would age into the survey in future waves, referred to hereafter as shadow youths (80.2% response rate). At wave 4 (December 2016 to January 2018), a replenishment sample added 3739 youths aged 12 to 17 years (70.6% response rate) to the surveys and identified 4294 shadow youths aged 10 to 11 years (78.7% response rate) who would age into future surveys. Follow-up interviews for all respondents were conducted approximately 1 year after the previous survey. The annual follow-up response rates for the wave 1 youth cohort were 87.3%, 83.3%, 79.5%, 74.6%, and 72.3% for the 5 next annual waves, respectively. The annual follow-up response rates for the wave 4 cohort (which included both the wave 1 cohort and replenishment samples) were 89.1% and 83.5% for the next 2 waves, respectively. All surveys included youth providing assent after a parent or legal guardian provided informed consent and were overseen by the Westat Institutional Review Board. We followed the Strengthening the Reporting of Observational Studies in Epidemiology (STROBE) reporting guideline for cohort studies.

Our analytical sample consisted of pooled observations of youth (aged 12-17 years at their baseline and follow-up surveys) who were past-30-day cigarette smokers at any annual wave, who had completed a survey preceding their report of current smoking, and who responded to question on current use of menthol cigarettes (waves 1-2, 427 respondents; waves 2-3, 292 respondents; waves 3-4, 271 respondents; waves 4-4.5, 277 respondents; waves 4.5-5, 225 respondents). We excluded youth who completed the adolescent survey at 1 wave but aged into an adult survey at a subsequent wave (7469 respondents), because survey measures on key study variables differed between the youth and adult sample. The schema of this analytical strategy is presented in Table 1.

**Table 1.** Timing of Measurements in the Cohorts of Youth Cigarette Smokers in the Population Assessment of Tobacco and Health Study, 2013-2019

Cohort	Survey wave					
	1	2	3	4	4.5	5
1	Menthol use and covariates	Menthol use and outcomes	NA	NA	NA	NA
2	NA	Menthol use and covariates	Menthol use and outcomes	NA	NA	NA
3	NA	NA	Menthol use and covariates	Menthol use and outcomes	NA	NA
4	NA	NA	NA	Menthol use and covariates	Menthol use and outcomes	NA
5	NA	NA	NA	NA	Menthol use and covariates	Menthol use and outcomes

Abbreviation: NA, not applicable.

We used a design-based approach to account for observations at multiple waves and the 396 respondents who contributed observations to multiple cohorts. Specifically, each cohort received a unique longitudinal survey weight and replicate weights at the respective time of observations, and these were assumed to represent a unique subsample of the population at each time point. Cohorts after wave 4 were weighted using the wave 4 cohort weights. Cohort membership was included in the propensity score analysis procedures to adjust for any effect from waves of assessment. This design allowed for covariates selected a priori to precede assessments of menthol and past-30-day cigarette use, for assessments of changes in smoking status and changes to and from menthol use, and allowed for shadow youth to age into the sample, thus increasing the analytical sample size.

### Measures

#### Smoking Frequency

Respondents were considered a current cigarette smoker if they had smoked in the 30 days before the survey. All current smokers were asked, “In the past 30 days, on how many days did you smoke cigarettes?” and provided a response ranging from 0 to 30 days. Like others,^19^ we defined frequent smoking as smoking on 20 or more of these 30 days.

#### Nicotine Dependence

The PATH Study used 7 items from the Wisconsin Inventory of Smoking Dependence Motives framework^20^ (see the eAppendix in the Supplement). The range of ND scores was 7 to 35, with 7 indicating no evidence of ND and included those who were not current smokers.

#### Menthol Use

Youth menthol use status was based on a yes response to the question, “In the past 30 days, were any of the cigarettes you smoked flavored to taste like menthol or mint?” Transitions to smoking either menthol or nonmenthol cigarettes were assessed among those who were nonsmokers at baseline but current smokers at follow-up. Respondents who reported nonmenthol cigarette use at 1 wave and menthol use at the next wave (or vice versa) were considered switchers. Respondents who reported menthol use at both sequential surveys were considered maintainers.

### Study Covariates

We included the following potential confounders assessed at the baseline survey: age, race and ethnicity, sex, symptoms of internalizing or externalizing mental health issues, time spent around smokers, grades in school, perceived harm of cigarettes, parent-perceived overall health, lifetime drug and alcohol use, past-30-day use of noncigarette tobacco products, the number of cigarettes smoked in entire life, the recency of the last smoked a cigarette, and the reference cohort number. Race and ethnicity were self-identified by the respondents through the survey and were analyzed in this study to evaluate associations between race and ethnicity and the likelihood of smoking menthol cigarettes, smoking frequency, and the level of ND. For analyses that included baseline current smokers, we added whether the respondent had a regular brand of cigarettes, the number of days the respondent smoked in the 30 days before the baseline survey, and the level of ND (see the eAppendix in the Supplement).

### Managing Missing Data

Missing data (eTable 1 in the Supplement) were imputed using the Amelia II algorithm in R statistical software version 4.0.4 (R Project for Statistical Computing) with 5 imputed data sets before IPTW and analysis procedures.^21^ Imputation diagnostics suggest that imputed values accurately estimated observed values (eFigure in the Supplement).

### Statistical Analysis

Weighted means, percentages, and 95% CIs were calculated using the longitudinal composite survey weights and replicate weights, which adjusted for the complex sampling design and longitudinal dropout and were calculated using balanced repeated replication with the Fay adjustment (ρ = 0.3).^22^ To calculate propensity scores, separate logistical models were fitted to estimate 5 comparisons of menthol use: (1) menthol use vs not at follow-up, (2) maintained menthol use vs maintained no menthol use, (3) switched to menthol vs maintained no menthol use, (4) switched from menthol vs maintained menthol use, and (5) transitioned from not smoking to menthol use vs transitioned from not smoking to nonmenthol use. Variables in these models included all potential confounders and, as recommended,^23^ the composite survey weights. Stabilized inverse propensity score weights were computed with the resulting propensity scores.^24^ Diagnostics of the resulting distributions of stabilized weights suggested no evidence of nonpositivity or of misspecification of the propensity score model because all means were near 1.0 and there were no extreme observations across any of the imputed data sets (eTable 2 in the Supplement).^25^ The resulting stabilized weights were multiplied by the composite survey weights to form a final weight for subsequent balance assessments and effect estimation.^23^

To assess balance between the menthol use groups, weighted standardized mean differences (SMDs) of each potential confounder were computed between the exposed and unexposed groups before and after, including the stabilized inverse propensity scores with the composite weights.^26^ We chose a conventional threshold of the absolute value of the SMD below 0.2 as an indicator of good balance.^27^ The association of menthol use with smoking frequency and ND was estimated via IPTW-adjusted mean differences (aMDs), adjusted risk ratios (aRRs), and corresponding 95% CIs. ND scores were log-transformed to express mean differences on the ratio scale. Significance was assessed by the inclusion or exclusion of the null (0 for differences and 1 for ratios) in the 95% CI. All analyses were performed using R statistical software version 4.0.4 (R Project for Statistical Computing). Data were analyzed from December 2021 to March 2022.

## Results

There were a total of 1492 observations from 1096 US youth cigarette smokers, of whom 49.4% (95% CI, 46.0%-52.8%) were female, 67.2% (95% CI, 64.3%-70.1%) were non-Hispanic White, and 28.7% (95% CI, 26.4%-31.1%) were aged 12 to 14 years at their baseline survey (all percentages are weighted) (Table 2). Youth cigarette smokers used menthol cigarettes at their follow-up survey in 56.3% (95% CI, 53.1%-59.4%) of observations (Table 3). The most prevalent pattern of use was transitioning from not smoking at all at baseline to using menthol at the follow-up survey (33.2%; 95% CI, 30.6%-35.9%). Youth who used menthol at their follow-up survey smoked a mean of 12.8 days (95% CI, 11.8-13.7 days), compared with 9.4 days (95% CI, 8.4-10.3 days) among nonmenthol users, and 34.1% (95% CI, 30.3%-37.8%) were frequent smokers, compared with 22.5% (95% CI, 18.8%-26.3%) among nonmenthol users. On the 7 to 35 ND scale, youth who used menthol at their follow-up survey tended to be more nicotine dependent (mean ND score, 13.8; 95% CI, 13.1-14.5) than nonmenthol smokers (mean ND score, 12.3; 95% CI, 11.8-12.9). Additional differences were also observed across transitions in use (Table 3).

**Table 2.** Description of the Sample of Youth Past-30-Day Cigarette Users in the Population Assessment of Tobacco and Health Study, 2013-2019

Variable	Weighted %, mean (95% CI)^a^
Age 12-14 y	28.7 (26.4-31.1)
Race and ethnicity	
Hispanic	17.9 (15.9-19.8)
Non-Hispanic Black	6.8 (4.8-8.8)
Non-Hispanic White	67.2 (64.3-70.1)
Non-Hispanic other^b^	8.1 (6.5-9.8)
Sex	
Female	49.4 (46.0-52.8)
Male	50.6 (47.2-54.0)
Problem symptoms, No. of symptoms	
Internalizing (0-4 symptoms)	2.4 (2.4-2.5)
Externalizing (0-7 symptoms)	3.4 (3.2-3.5)
Time spent around smokers, h	11.3 (9.5-13.0)
Any household tobacco use	60.5 (56.4-64.6)
Perceived harm of cigarette smoking, score of 1 (no harm) to 4 (a lot of harm)	3.5 (3.5-3.6)
Grades in school (mostly Bs and higher)	41.9 (38.4-45.5)
Parent-reported overall health, score of 1 (excellent) to 5 (poor)	1.8 (1.8-1.9)
Any lifetime drug use	57.2 (53.7-60.7)
Any baseline past-30-d noncigarette tobacco use	37.9 (35.3-40.5)
Lifetime cigarette consumption, score of 0 (never smoked) to 7 (≥100 cigarettes)	3.1 (2.9-3.2)
Time since last cigarette smoked, score of 1 (today) to 8 (never smoked)	4.7 (4.5-4.8)
Cohort	
1	27.4 (25.0-29.7)
2	21.3 (19.0-23.6)
3	20.9 (18.9-23.0)
4	16.3 (14.7-17.8)
5	14.1 (12.2-16.1)
Had a regular brand of cigarettes	21.7 (19.2-24.2)
Days smoked in previous 30 d (range, 0-30 d), No.	5.3 (4.7-5.9)
Nicotine dependence score of 7 (no evidence of dependence) to 35	10.2 (9.8-10.5)

^a^ Estimates are calculated on the full sample using the composite survey weights provided by the Population Assessment of Tobacco and Health Study. All measures are assessed at the respective cohort’s baseline survey.

^b^ Refers to all other races including Asian, Native American, Pacific Islander, and multiracial.

**Table 3.** Menthol Use, Frequency of Cigarette Smoking, and Level of Nicotine Dependence Among Youth Past-30-Day Cigarette Users in the Population Assessment of Tobacco and Health Study, 2013-2019

Variable	Respondents, No. (N = 1492)	Mean (95% CI)^a^			
		Menthol use status, %	Days smoked cigarettes in previous 30 d, No.	Smoked cigarettes on ≥20 d in previous 30 d, %	Nicotine dependence score (range, 7-35)
Overall menthol use at follow-up					
Yes	848	56.3 (53.1-59.4)	12.8 (11.8-13.7)	34.1 (30.3-37.8)	13.8 (13.1-14.5)
No	644	43.7 (40.6-46.9)	9.4 (8.4-10.3)	22.5 (18.8-26.3)	12.3 (11.8-12.9)
Transitions					
Maintained menthol use					
Yes	261	16.9 (14.6-19.3)	18.1 (16.4-19.8)	54.3 (47.0-61.5)	16.2 (15.0-17.4)
No	114	7.8 (6.0-9.6)	16.0 (13.2-18.8)	45.7 (34.3-57.2)	14.4 (12.8-15.9)
Switched					
From menthol	120	8.1 (6.9-9.3)	13.0 (11.2-14.8)	32.3 (23.6-41.0)	15.5 (14.3-16.6)
To menthol	93	6.1 (4.9-7.2)	14.0 (11.2-16.7)	39.4 (28.0-50.9)	14.7 (12.9-16.5)
No smoking to menthol use	494	33.2 (30.6-35.9)	9.8 (8.9-10.7)	22.8 (19.3-26.3)	12.4 (11.8-13.0)
No smoking to nonmenthol use	410	27.8 (25.0-30.7)	6.5 (5.6-7.3)	13.2 (10.3-16.1)	10.8 (10.3-11.4)

^a^ Estimates are calculated on the full sample using the composite survey weights provided by the Population Assessment of Tobacco and Health Study.

Overall, menthol cigarette users were similar to nonmenthol cigarette users (using the absolute value of the SMD >0.2 as an a priori threshold for imbalance), but several of the transition groups were imbalanced on some of the covariates when weighted with only the composite survey weights (Table 4). For example, compared with maintainers of nonmenthol use, maintainers of menthol use had more symptoms of externalizing problem behaviors (SMD = 0.32), spent more time around other smokers (SMD = 0.27), perceived that a greater harm could result from smoking (SMD = 0.28), and were more frequent smokers (SMD = 0.24). IPTW improved the comparability of these comparisons for every variable we assessed, including the propensity scores, resulting in SMDs that were closer to 0 than when only the composite weights were used. The final IPTW comparisons were well balanced, because the absolute values of the SMDs for all variables and the propensity scores were below the conventional threshold of SMD less than 0.2 for all comparisons.

**Table 4.** Standardized Mean Differences Showing the Balance Improvement Obtained by Weighting With Propensity Scores for Use and Transitions in Use of Menthol Among Youth Past-30-Day Cigarette Users in the Population Assessment of Tobacco and Health Study, 2013-2019^a^

Characteristics measured at baseline	Standardized mean differences									
	Overall menthol use at follow-up vs nonmenthol use at follow-up		Maintained menthol vs maintained nonmenthol		Switched from menthol vs maintained menthol		Switched to menthol vs maintained nonmenthol		Not smoking to menthol vs not smoking to nonmenthol	
	Composite	IPTW	Composite	IPTW	Composite	IPTW	Composite	IPTW	Composite	IPTW
Propensity score	0.35^b^	−0.02	0.79^b^	0.02	0.57^b^	−0.06	0.78^b^	−0.05	0.41^b^	−0.01
Age 12-14 y	−0.01	0.03	0.06	−0.01	0.10	−0.02	0.17	−0.11	0.01	0.05
Race and ethnicity										
Hispanic	0.06	−0.01	0.08	−0.01	0.07	−0.02	0.15	−0.01	0.08	−0.03
Non-Hispanic Black	0.00	0.02	0.10	−0.02	−0.03	−0.04	0.11	−0.05	−0.04	0.03
Non-Hispanic White	0.06	−0.04	0.10	−0.07	−0.09	0.13	0.05	−0.07	0.04	−0.01
Non-Hispanic other^c^	−0.08	0.02	−0.17	0.06	0.02	−0.05	−0.21^b^	0.08	−0.07	0.01
Female sex	0.12	0.01	0.18	0.05	−0.26^b^	−0.01	0.24^b^	0.04	0.04	−0.01
Problem symptoms										
Internalizing	0.03	−0.03	0.11	0.01	−0.15	0.06	0.07	0.00	−0.04	−0.04
Externalizing	0.14	0.00	0.32^b^	0.04	−0.19	0.05	0.18	−0.02	0.08	0.00
Time spent around smokers	0.10	0.02	0.27^b^	0.07	−0.18	−0.01	0.07	−0.11	0.00	0.03
Any household tobacco use	0.01	−0.01	−0.07	0.00	−0.14	−0.01	−0.22^b^	−0.05	0.00	−0.01
Perceived harm of cigarette smoking, score of 1 (no harm) to 4 (a lot of harm)	0.11	0.02	0.28^b^	0.08	−0.08	0.07	0.28^b^	0.05	0.09	0.00
Grades in school (mostly Bs or higher)	−0.03	−0.01	0.14	−0.02	−0.21^b^	−0.06	0.13	0.00	−0.14	−0.05
Parent-reported overall health, score of 1 (excellent) to 5 (poor)	0.03	−0.03	0.17	−0.01	−0.19	0.09	−0.14	−0.14	−0.03	−0.01
Any lifetime drug use	0.05	0.02	0.18	0.06	−0.22^b^	−0.03	0.00	0.00	−0.03	0.02
Any baseline past 30 d noncigarette tobacco use	0.07	0.01	0.16	0.06	−0.07	−0.03	0.04	0.04	0.02	0.00
Lifetime cigarette consumption, score of 0 (never smoked) to 7 (≥100 cigarettes)	0.09	−0.03	0.05	−0.07	−0.12	0.03	−0.29^b^	−0.02	0.07	−0.04
Time since last cigarette smoked, score of 1 (today) to 8 (never smoked)	−0.06	0.03	0.07	0.10	0.11	−0.04	0.34^b^	0.05	0.02	0.03
Cohort										
1	−0.11	0.03	−0.12	0.02	0.15	−0.01	−0.06	−0.02	−0.11	0.05
2	0.13	−0.03	0.06	−0.01	−0.16	0.06	0.15	−0.11	0.12	−0.03
3	−0.08	−0.01	0.08	0.01	0.01	−0.01	−0.06	0.01	−0.12	−0.03
4	0.10	0.03	−0.12	−0.04	0.02	−0.04	−0.18	0.12	0.21^a^	0.05
5	−0.02	−0.03	0.12	0.02	−0.04	−0.02	0.12	0.02	−0.09	−0.05
Had a regular brand of cigarettes	0.06	−0.04	0.01	−0.06	−0.09	0.05	−0.25^b^	−0.07	NA	NA
Days smoked in previous 30 d, No.	0.09	−0.04	0.24^b^	−0.06	−0.13	0.08	−0.30^b^	−0.07	NA	NA
Nicotine dependence score of 7 (no evidence of dependence) to 35	0.07	−0.02	0.19	0.07	0.01	0.03	−0.11	0.00	NA	NA

Abbreviations: IPTW, inverse probability of treatment weights; NA, not applicable.

^a^ Each value indicates the standardized mean difference of the covariate between menthol use and transitions in use calculated either by using original data sets and weights (composite) or using the IPTW.

^b^ The absolute value of standardized mean differences that were 0.2 or less indicate good balance vs values greater than 0.2.

^c^ Refers to all other races including Asian, Native American, Pacific Islander, and multiracial.

In the Figure, we present the IPTW-adjusted associations between menthol use and transitions in use with the measures of smoking frequency and ND. Menthol use was associated with smoking on 2.8 additional days (aMD; 95% CI, 1.4 to 4.2 days), 38% higher risk of being frequent smokers (aRR, 1.38; 95% CI, 1.12 to 1.70), and 8% higher ND scores (aMD, 1.08 log-scaled; 95% CI, 1.01 to 1.14). Similarly, among those who switched from not smoking to smoking (61% of the sample), menthol use was associated with smoking on 3.1 additional days (aMD; 95% CI, 1.9 to 4.2 days), 59% higher risk of being a frequent smoker (aRR, 1.59; 95% CI, 1.23 to 2.06), and 10% higher ND scores (aMD, 1.09; 95% CI, 1.02 to 1.17). Switching from menthol (vs maintaining menthol use) was associated with smoking on 3.6 fewer days (aMD; 95% CI, −6.3 to −0.9 days), 47% lower risk of being a frequent smoker (aRR, 0.68; 95% CI, 0.50 to 0.92), and 3% lower ND scores (aMD, 0.97; 95% CI, 0.87 to 1.09).

**Figure.**
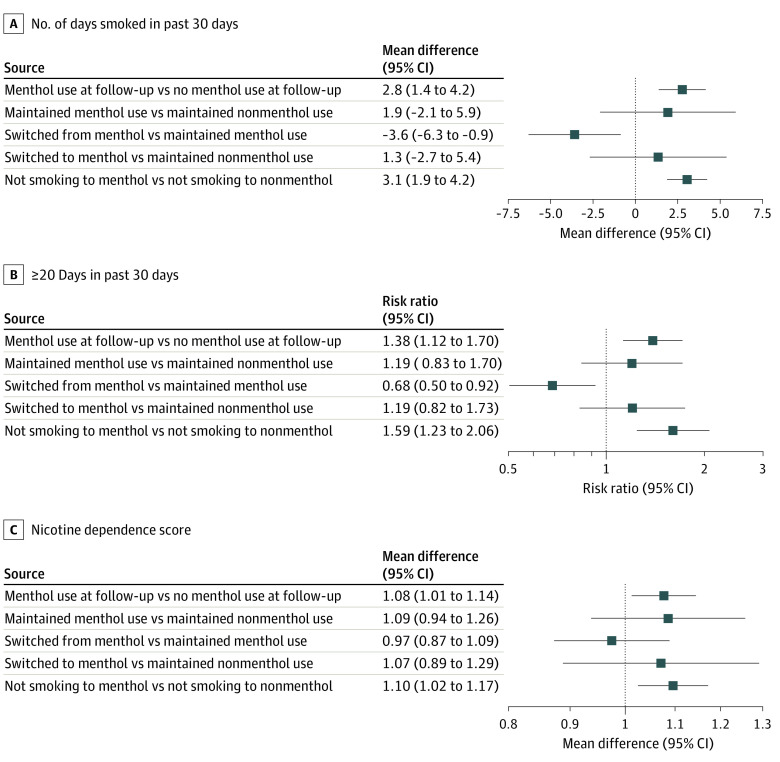
Frequency of Smoking in the Previous 30 Days, Risk of Smoking at Least 20 Days in the Previous 30 Days, and Level of Nicotine Dependence Among Youth Past-30-Day Cigarette Users in the Population Assessment of Tobacco and Health Study, 2013-2019 All estimates were calculated using inverse probability of treatment weighting. Mean differences for nicotine dependence were expressed on the ratio scale.

## Discussion

In this nationally representative cohort study, the majority of cigarette smokers aged 12 to 17 years used menthol cigarettes. IPTW considerably improved the comparability of menthol use groups across all potential confounding variables, resulting in a well-balanced analytical sample. The IPTW-adjusted effect estimates suggested that menthol use was associated with increased smoking frequency and ND but that switching from menthol to nonmenthol cigarettes was associated with reduced smoking frequency and ND.

These findings further support the conclusions of a recent review of observational research on youth menthol use,^14^ as well as previous longitudinal studies.^15,16,17^ We used 6 waves of PATH Study data and identified 5 cohorts each with 2 waves of data. By combining the experience of these cohorts, we had sufficient sample size to compare transitions in flavor use in a target trial framework^28^ that mirrors how a randomized clinical trial might assess the impacts of menthol use if it were ethical to conduct one among youth. These methodological improvements meant that our study had considerably more power to address hypotheses in the youth sample than did the study by Villanti et al,^18^ which found no association between youth menthol use and subsequent smoking frequency or ND in the first 4 waves of the PATH study. However, our findings were consistent with those that Villanti et al^17^ reported for the young adult (aged 18-24 years) sample. We chose not to include youth who aged into the adult sample because of differences in survey questions between the PATH Study youth and adult surveys; however, the comparison to Villanti et al^18^ suggests that also including young adults could have increased the magnitude of the association in our study. These findings warrant future studies that use consistent measurements of menthol and tobacco use over longer durations as youth transition to young adulthood and potentially develop more established patterns of tobacco use.

Our use of IPTW was also a methodological advancement that allowed us to ensure that our menthol and nonmenthol user groups were as comparable as possible on factors that might be related to an individual’s smoking frequency or level of ND and also related to their self-selected use of menthol (eg, age), sometimes referred to as confounding variables.^29,30^ The results suggest that our comparisons were well balanced, improving confidence that the estimates have adjusted for the potential confounding variables that were measured in the PATH Study and included in our analyses.

### Limitations

Some limitations of our study should be noted. First, although the analysis of switching to or from menthol can be viewed as a strength, the extent to which this unprompted change in behavior models a forced change in behavior, as would be the case with a menthol ban or a randomized clinical trial, is unclear. Second, although our measurement of menthol use at 2 time points provides an indication of the duration of menthol use, the quantity of menthol cigarettes used at each time point could not be determined, making it unclear whether respondents used menthol exclusively. It is also possible that menthol users switched to and from menthol in the time between surveys, and this could not be accounted for. Third, although some potentially confounding variables (eg, greater lifetime cigarette use) were identified and accounted for with IPTW, as with any observational study it is possible that there were additional unmeasured confounding variables or that residual confounding could explain the observed association. Fourth, it is possible that a few of the respondents’ use of nonmenthol was an artifact of state or local jurisdiction enactment of menthol policies, which primarily occurred after 2019,^31^ and could not be considered because geographical data are censored in the PATH Study.

## Conclusions

The findings of this cohort study suggest that the addition of menthol to cigarettes is associated with increased smoking frequency and ND among youth. As such, these results provide strong support for recommendations to ban menthol as a characterizing flavor in cigarettes as a protection for youth.^4^

## Notes

Supplement. **eAppendix.** Supplemental Information on Survey Measures **eTable 1.** Description of Missingness Among the Baseline Characteristics of the Sample of Youth Past 30-Day Cigarette Users in the PATH Study, USA, 2013-2019 **eFigure.** Model Diagnostics for Ordinal and Continuous Data With Missing Values **eTable 2.** Distributions of Stabilized Weights Suggested No Evidence of Nonpositivity or of Misspecification of the Propensity Score Model as All Means Were Near 1 and There Were No Extreme Observations
eReferences
