# Supplementary material for: Use of Menthol Cigarettes, Smoking Frequency, and Nicotine Dependence Among US Youth
Source: JAMA Netw Open. 2022 Jun 6;5(6):e2217144. doi: 10.1001/jamanetworkopen.2022.17144 (PMC9171563; doi:10.1001/jamanetworkopen.2022.17144)
Supplement: Supplement. — eAppendix. Supplemental Information on Survey Measures eTable 1. Description of Missingness Among the Baseline Characteristics of the Sample of Youth Past 30-Day Cigarette Users in the PATH Study, USA, 2013-2019 eFigure. Model Diagnostics for Ordinal and Continuous Data With Missing Values eTable 2. Distributions of Stabilized Weights Suggested No Evidence of Nonpositivity or of Misspecification of the Propensity Score Model as All Means Were Near 1 and There Were No Extreme Observations eReferences [file jamanetwopen-e2217144-s001.pdf]

Supplemental Online Content

Leas EC, Benmarhnia T, Strong DR, Pierce JP. Use of menthol cigarettes, smoking frequency, and nicotine dependence among US youth. *JAMA Netw Open*. 2022;5(6):e2217144.
doi:10.1001/jamanetworkopen.2022.17144

eAppendix. Supplemental Information on Survey Measures
eTable 1. Description of Missingness Among the Baseline Characteristics of the Sample of Youth Past 30-Day Cigarette Users in the PATH Study, USA, 2013-2019
eFigure. Model Diagnostics for Ordinal and Continuous Data With Missing Values
eTable 2. Distributions of Stabilized Weights Suggested No Evidence of Nonpositivity or of Misspecification of the Propensity Score Model as All Means Were Near 1 and There Were No Extreme Observations
eReferences

This supplemental material has been provided by the authors to give readers additional information about their work.

eAppendix. Supplemental Information on Survey Measures

Socio-demographics. Questions assessing age, sex and race/ethnicity were administered at all waves. Missing data on age, sex, race, and Hispanic ethnicity were logically assigned from other survey data as described in the PATH Study User Guide.[1] Educational status was also obtained and since college attenders typically have a much higher successful cessation rate than other education groups this variable will be dichotomized (college graduate vs. less than college graduate).[2]

Symptoms of Internalizing or Externalizing Mental Health Issues. Mental health and substance use problems were assessed using scales from the Global Appraisal of Individual Needs–Short Screener.[3] The “internalizing” subscale included 4 items of depressive and anxiety symptoms. The “externalizing” subscale included experience with 5 conduct and behavioral items. Respondents were scored on how many items they had experienced in the past month or past 2 to 12 months.[4,5]

Time Around Smokers. Respondents were asked, “during the past seven days, about how many hours were you around others who were smoking [whether or not you were smoking yourself]? Include time in your home, in a car, at work, or outdoors.” Responses were recorded in hours.

Grades in School. All parents were asked “How would you describe how {Child’s first name} has performed at school in the past 12 months? Would you say {Child’s first name}’s grades are...” with response options given from “Mostly A’s” to “Mostly F’s” as well as an option for whether the child’s school was ungraded. We categorized this as either the child having mostly A’s, A’s and B’s, or mostly B’s or not.

Perceived Harm of Cigarettes. Respondents were asked, “How harmful do you think cigarettes are to health?” with responses "not at all harmful,” “slightly harmful,” “somewhat harmful,” “very harmful,” “extremely harmful.”

© 2022 Leas EC et al. *JAMA Network Open.*

Parent-perceived overall health. Parents recorded their perceived overall health of their child by responding to the question: “in general, would you say {Child’s first name}’s overall health is….” with the response options “Excellent,” “Very Good,” “Good,” “Fair,” “Poor.”

Lifetime Use of Drugs or Alcohol. Ever use was ascertained for alcohol, marijuana, as well as for misuse of prescription drugs (i.e., Ritalin/ Adderall, painkillers, sedatives, tranquilizers), cocaine or crack, methamphetamine or speed, heroin, inhalants, solvents, and hallucinogens by a series of questions: “Have you ever used [substance]?” Youth who reported ever use of any of these were classified as “any use;” all others were classified as “no use.”

Tobacco Use Status. Participants were asked a series of questions about the types of tobacco products they used. Details of questions and responses are presented in Kasza et al.[6] We adjust for “current” (i.e., past-30 day) use of cigars, cigarillos, e-cigarettes, hookah, snus and smokeless tobacco as sources of potential confounding. Youth who reported established use of any tobacco product in other than cigarettes were classified as “any use;” while all others were classified as “no use.”

Lifetime Cigarette Use. All youth who and ever smoked a cigarette, even one or two puffs were asked “How many cigarettes have you smoked in your entire life? A pack usually has 20 cigarettes in it” with the response options “1 or more puffs but never a whole cigarette,” “1 cigarette”, “2 to 10 cigarettes (about ½ pack total)”, “11 to 20 cigarettes (about ½ pack to 1 pack)”, “21 to 50 cigarettes (more than 1 pack but less than 3 packs)”, “51 to 99 cigarettes (more than 2 ½ packs but less than 5 packs)”, and “100 or more cigarettes (5 packs or more).” We used these categories and created a category of “never smoked.”

Recency of Last Cigarette Used. All youth were asked “have you ever tried cigarette smoking, even one or two puffs?” with response option “yes” or “no” and those indicating that they had ever smoked were later asked “when was the last time you smoked a cigarette, even one or two puffs?” with response options ranging from “earlier today,” “not today but sometime in the past 7 days,” “not in the past 7 days but sometime in the past 30 days,” “not in the past 30

days but sometime in the past 6 months," "not in the past 6 months but sometime in the past year," "1 to 4 years ago," and "5 or more years ago." Respondents were considered a current cigarette smoker if they had smoked in the 30 days prior to the time of the survey and in matching procedures we used all response options and created a category of "never smoked."

Cohort Number. Since samples will be pooled across waves, we also adjust for any potential confounding effects due to the years the cohorts were surveyed.

Regular Brand of Cigarettes. All respondents who have smoked a cigarette in the past 30 days, and had smoked more than 10 cigarettes in their lifetime were asked "Do you have a regular brand of cigarettes that you usually smoke?" with responses being either "yes" or "no." We categorized this as either having a regular brand or not, with those who had not smoked ≤10 cigarettes in their lifetime being assigned as not having a regular brand.

Smoking frequency: All youth who were current smokers were asked "In the past 30 days, on how many days did you smoke cigarettes?" and provided a numeric response from 0 to 30 days. A value of 0 was assigned to respondents who were non-smokers at baseline.

Nicotine dependence: The PATH Study used the Wisconsin Inventory of Smoking Dependence Motives framework[7] and queried 7 domains on both the adolescent and adult questionnaires with 5 response options: "1 = Not true of me at all," "2," "3," "4," "5 = Extremely true of me." The questions were: "I find myself reaching for [product] without thinking about it;" "I frequently crave [product];" "My [product] use is out of control;" "I usually want to use [product] right after I wake up;" "Using [product] really helps me feel better if I've been feeling down;" "Using [product] helps me think better;" "I would feel alone without my [product]." For single product users' nicotine dependence refers to their product. For dual cigarette and e-cigarette users' nicotine dependence was taken from the cigarette items. For users of multiple products nicotine dependence was assessed from a generalized "tobacco" product rather than have repeated items for each product. The range of nicotine dependence scores is 7-35 with 7

© 2022 Leas EC et al. *JAMA Network Open.*

indicating no evidence of dependence. Respondents who were not current smokers at baseline were assigned the lowest level of dependence.

eTable 1. Description of Missingness Among the Baseline Characteristics of the Sample of Youth Past 30-Day Cigarette Users in the PATH Study, USA, 2013-2019

Variable	% Missing
Age (12-14 years vs. 15-17 years)	0.0%
Non-Hispanic Black (Yes vs. No)	0.1%
Hispanic (Yes vs. No)	0.1%
Non-Hispanic White (Yes vs. No)	0.1%
Non-Hispanic Other (Yes vs. No)[a]	0.1%
Sex (Female vs. Male)	0.0%
Internalizing Problem Symptoms (Number of Symptoms)	2.7%
Externalizing Problem Symptoms (Number of Symptoms)	3.2%
Hours Spent Around Smokers (Hours)	3.8%
Household Tobacco Use (Any vs. None)	1.1%
Perceived Harm of Cigarette Smoking (No Harm to a Lot of Harm, 1-4)	0.5%
Grades in School (≥Mostly B's vs. <Mostly B's)	1.1%
Parent-Reported Overall Health (Excellent to Poor, 1-5)	0.7%
Lifetime Drug Use (Any vs. None)	0.8%
Past 30 Day Non-cigarette Tobacco Use (Any vs. None)	2.4%
Lifetime Cigarette Consumption (Never smoked to ≥100 cigarette, 0-7)	1.1%
Time Since Last Cigarette Smoked (Today to Never Smoked, 1-8)	2.1%
Cohort 1 (Yes vs. No)	0.0%
Cohort 2 (Yes vs. No)	0.0%
Cohort 3 (Yes vs. No)	0.0%
Cohort 4 (Yes vs. No)	0.0%
Cohort 5 (Yes vs. No)	0.0%
Had a Regular Brand of Cigarettes (Yes vs. No)	0.1%
Number of Days Smoked in Previous 30 Days (Days)	1.1%
Nicotine Dependence Score (7-35)	1.0%

Note: All measures are presented assessed at baseline.

[a]Non-Hispanic Other refers to all other races, including Asian, Native American, Pacific Islander, and multiracial.

© 2022 Leas EC et al. *JAMA Network Open.*

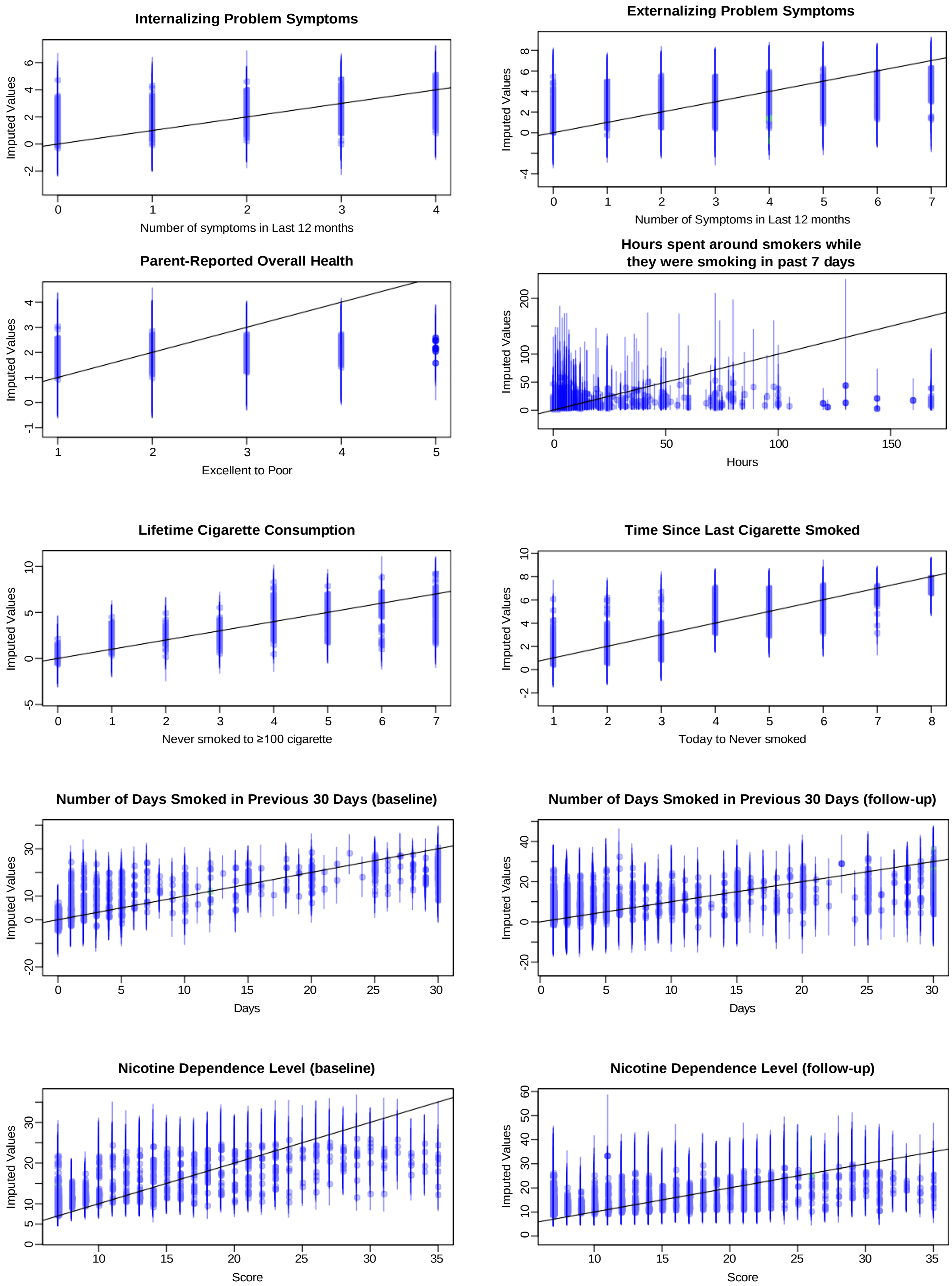

eFigure. Model Diagnostics for Ordinal and Continuous Data With Missing Values. Notes: Values in the above figure assess how accurate the imputed data are by sequentially treating the observed values as if they were missing and generating several hundred imputed values (allowing the construction of confidence intervals) for them as if they were in fact missing. If the true and observed values' confidence intervals cross the x = y line (imputation perfectly agrees with actual data) then the imputation model is doing well. Further details on this diagnostic method, known as overimputation, are available in Blackwell, Honaker and King 2015.[8] Imputed values were predicted using the Amelia II described by Honaker, King and Blackwell 2011[9]

© 2022 Leas EC et al. *JAMA Network Open.*

eTable 2. Distributions of Stabilized Weights Suggested No Evidence of Nonpositivity or of Misspecification of the Propensity Score Model as All Means Were Near 1 and There Were No Extreme Observations.

Analytical Comparison	Imputation	n	Mean	CV	P1	P99	Min	Max
Menthol Use vs. Non-menthol Use	1	1492	1.00	0.20	0.70	1.69	0.61	2.17
	2	1492	1.00	0.19	0.71	1.66	0.61	2.12
	3	1492	1.00	0.20	0.71	1.71	0.62	2.10
	4	1492	1.00	0.20	0.71	1.69	0.60	2.13
	5	1492	1.00	0.19	0.70	1.67	0.63	2.13
Maintained Menthol vs. Maintained Non-Menthol	1	375	0.99	0.39	0.42	2.67	0.38	3.06
	2	375	0.99	0.39	0.44	2.58	0.38	2.96
	3	375	0.99	0.39	0.42	2.58	0.37	3.24
	4	375	1.00	0.40	0.42	2.60	0.38	3.28
	5	375	0.99	0.38	0.43	2.49	0.38	3.10
Switched from Menthol vs. Maintained Menthol	1	381	1.00	0.30	0.52	2.30	0.44	2.93
	2	381	1.00	0.33	0.48	2.31	0.43	3.19
	3	381	1.00	0.32	0.51	2.48	0.43	3.00
	4	381	1.00	0.33	0.49	2.46	0.43	3.07
	5	381	1.01	0.33	0.51	2.43	0.44	3.45
Switched to Menthol vs. Maintained Non-menthol	1	207	1.01	0.51	0.58	3.20	0.53	4.21
	2	207	1.01	0.51	0.58	2.93	0.54	4.42
	3	207	1.01	0.48	0.57	3.07	0.56	4.20
	4	207	1.01	0.48	0.57	3.17	0.55	4.27
	5	207	1.01	0.51	0.57	3.08	0.54	4.61
Not Smoking to Menthol vs. Not Smoking to Non-menthol	1	904	1.00	0.21	0.69	1.68	0.65	2.23
	2	904	1.00	0.21	0.70	1.68	0.65	2.21
	3	904	1.00	0.23	0.68	1.76	0.64	2.33
	4	904	1.00	0.22	0.69	1.72	0.65	2.30
	5	904	1.00	0.22	0.68	1.73	0.63	2.20

CV = Coefficient of Variation; P1 = 1st Percentile; P99 = 99th Percentile

© 2022 Leas EC et al. *JAMA Network Open.*

eReferences

1. Westat. PATH Study Public Use Files User Guide. Published 2017. Accessed January 31, 2018. https://goo.gl/B2uj9G

2. Heckman JJ, Humphries JE, Veramendi G. Returns to Education: The Causal Effects of Education on Earnings, Health and Smoking. National Bureau of Economic Research; 2016. doi:10.3386/w22291

3. McDonell MG, Comtois KA, Voss WD, Morgan AH, Ries RK. Global Appraisal of Individual Needs Short Screener (GSS): psychometric properties and performance as a screening measure in adolescents. Am J Drug Alcohol Abuse. 2009;35(3):157-160. doi:10.1080/00952990902825421

4. Conway KP, Green VR, Kasza KA, et al. Co-occurrence of tobacco product use, substance use, and mental health problems among youth: Findings from wave 1 (2013-2014) of the population assessment of tobacco and health (PATH) study. Addict Behav. 2018;76:208-217. doi:10.1016/j.addbeh.2017.08.009

5. Green VR, Conway KP, Silveira ML, et al. Mental Health Problems and Onset of Tobacco Use Among 12- to 24-Year-Olds in the PATH Study. J Am Acad Child Adolesc Psychiatry. 2018;57(12):944-954.e4. doi:10.1016/j.jaac.2018.06.029

6. Kasza KA, Ambrose BK, Conway KP, et al. Tobacco-Product Use by Adults and Youths in the United States in 2013 and 2014. New England Journal of Medicine. 2017;376(4):342-353. doi:10.1056/NEJMsa1607538

7. Smith SS, Piper ME, Bolt DM, et al. Development of the Brief Wisconsin Inventory of Smoking Dependence Motives. Nicotine Tob Res. 2010;12(5):489-499. doi:10.1093/ntr/ntq032

8. Blackwell M, Honaker J, King G. A Unified Approach to Measurement Error and Missing Data: Details and Extensions. Sociological Methods & Research. 2017;46(3):342-369. doi:10.1177/0049124115589052

9. Amelia II: A Program for Missing Data | Honaker | Journal of Statistical Software. doi:10.18637/jss.v045.i07

© 2022 Leas EC et al. *JAMA Network Open.*
